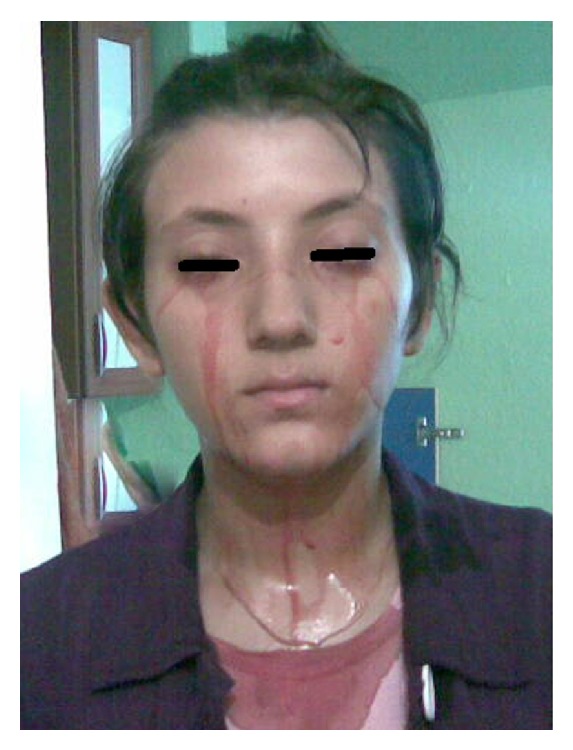# Idiopathic Bilateral Bloody Tearing

**DOI:** 10.1155/2015/692382

**Published:** 2015-01-22

**Authors:** Emrullah Beyazyıldız, Yasemin Özdamar, Özlem Beyazyıldız, Hasan Yerli

**Affiliations:** ^1^Department of Ophthalmology, Samsun Research and Training Hospital, Kıranköy Mevkii, İlkadım, 55100 Samsun, Turkey; ^2^Department of Ophthalmology, Ulucanlar Research and Training Hospital, 06030 Ankara, Turkey; ^3^Department of Radiology, University of Baskent, Zubeyde Hanim Research and Training Hospital, 35540 İzmir, Turkey

## Abstract

Bloody tear is a rare and distinct clinic phenomenon. We report a case presenting with the complaint of recurrent episodes of bilateral bloody tearing. A 16-year-old girl presented to our clinic with complaint of bloody tearing in both eyes for 3 months. Bloody tearing was not associated with her menses. A blood-stained discharge from the punctum was not observed during the compression of both nasolacrimal ducts. Nasolacrimal passage was not obstructed. Imaging studies such as dacryocystography and gradient-echo magnetic resonance imaging (MRI) of nasolacrimal canal were normal. Intranasal endoscopic evaluation was normal. We collected samples from bloody tears two times and pathological examination was performed. Pathological analysis showed lots of squamous cells and no endometrial cells; dysplastic cells were found. Further evaluations for underlying causes were unremarkable. No abnormalities were found in ophthalmologic, radiologic, and pathologic investigations. This condition is likely a rare abnormality and the least recognized aetiology for the idiopathic phenomenon.

## 1. Introduction

Bloody tear is a rare and distinct clinic phenomenon [1]. Although ocular and/or systemic abnormalities have been described for the etiology of bloody tear, etiologic causes have remained unclear in many cases [1]. In this study, we report a case presenting with the complaint of recurrent episodes of bilateral bloody tearing, where a wide investigation was needed to exclude significant conditions, ultimately leading to a diagnosis of an unknown bloody tearing.

## 2. Case Report

A 16-year-old girl presented to our clinic with complaint of bloody tearing on both eyes for 3 months (Figure 1). Her menarche started when she was 13 and her menstrual periods were normal. Bloody tearing was not associated with her menses. Medical history was negative for coagulopathy, bleeding diathesis, and hematologic abnormalities. She did not acknowledge any previous trauma. There was no clinically significant systemic or ocular history in her family. Her menstrual cycle was normal and she had no symptoms such as polymenorrhea, oligomenorrhea, menorrhagia, or metrorrhagia.

In ocular examination, her visual acuity was 20/20 in both eyes. Anterior segment findings, intraocular pressures, and fundus examinations were normal. Pupils were equal in size and reactive to light. There were not any other abnormalities including foreign body, vascular lesion, and laceration, on both eyelids, conjunctiva, cornea, and punctal region. The lacrimal gland was not enlarged or tender, and no discharge was noticed on compression. A blood-stained discharge from the punctum was not observed during the compression of both nasolacrimal ducts. Nasolacrimal passage was not obstructed. Imaging studies such as dacryocystography and gradient-echo magnetic resonance imaging (MRI) of nasolacrimal canal were normal. Intranasal endoscopic evaluation was normal. Then we collected samples from bloody tears two times and pathological examination was performed. Pathological analysis showed lots of squamous cells and no endometrial cells; dysplastic cells were found.

The patient was consulted to gynecology and hematology departments. Further investigation into possible causes included comprehensive bleeding diathesis screening. Normal results were obtained for platelet count, prothrombin time, activated partial thromboplastin time, blood coagulation panel, bleeding time, serum biochemistry, and complete blood cell count. Abdominal ultrasonography revealed an ovarian cyst in 1-2 cm dimensions. Further evaluations for underlying causes were unremarkable and she has been still followed up regularly.

## 3. Discussion

Bloody tearing has been rarely described and it can result from multiple disorders including hemangiomas, fibromas, inflammatory granulomas, hereditary hemorrhagic telangiectasia, malignant melanomas, nasolacrimal anomalies, lacrimal gland and sac tumors, nasolacrimal endometriosis, and hemophilia [2–5]. In the literature, several cases with bloody tearing have been reported. Though some patients have been associated with ocular and systemic disease, etiologic causes are unknown in many cases.

Hasner [2] reported two cases with bloody tearing: one, in which hemorrhage occurred from a conjunctival polyp in a man, and the other, which involved a 13-year-old girl with daily hemorrhage from the eyes. Their cases had no pathologic abnormalities. Author stated that a distinction of hemorrhages should be made between the lacrimal gland and conjunctiva. Ho et al. [1] reported four cases of unilateral idiopathic bloody tearing and the hemorrhages of their cases spontaneously resolved. In another study, Türkçüoğlu et al. [3] reported a case with bloody tearing. A 13-year-old girl with a history of cyclic bleeding, concomitant with menstruation, from the inferior punctum of her left eye was reported and no other ocular pathology was accompanying her symptoms. Gradient-echo MRI of the nasolacrimal canal during menstrual period was performed and it showed hypointense area with acute hemorrhage. Biopsy was not performed. They described this case as a “presumed nasolacrimal endometriosis.”

In our patient, there was bilateral bloody tear intermittently and irregularly, and it was not associated with her menses. No abnormalities were found in ophthalmologic, radiologic, and pathologic investigations. Detailed systemic evaluations were normal. The purpose of this paper is to report detailed ocular examinations and differential diagnosis of such a patient. This condition is likely a rare abnormality and the least recognized aetiology for the idiopathic phenomenon.

In conclusion, bloody tearing is an extremely rare finding. Although ophthalmologists have to exclude various diseases for idiopathic bloody tearing, diagnosis of bloody tearing remains a problem.

## Figures and Tables

**Figure 1:**